# Multimorbidity and quality of life after blast-related injury among US military personnel: a cluster analysis of retrospective data

**DOI:** 10.1186/s12889-020-08696-4

**Published:** 2020-04-28

**Authors:** Andrew J. MacGregor, James M. Zouris, Jessica R. Watrous, Cameron T. McCabe, Amber L. Dougherty, Michael R. Galarneau, John J. Fraser

**Affiliations:** 1 Medical Modeling, Simulation, and Mission Support Department, Naval Health Research Center, 140 Sylvester Road, San Diego, CA 92106 USA; 2 Leidos, Inc., 140 Sylvester Road, San Diego, CA 92106 USA; 3 Warfighter Performance Department, Naval Health Research Center, 140 Sylvester Road, San Diego, CA 92106 USA

**Keywords:** Blast injury, Combat, Deployment, Military, Multimorbidity, Quality of life

## Abstract

**Background:**

Blast injury emerged as a primary source of morbidity among US military personnel during the recent conflicts in Iraq and Afghanistan, and led to an array of adverse health outcomes. Multimorbidity, or the presence of two or more medical conditions in an individual, can complicate treatment strategies. To date, there is minimal research on the impact of multimorbidity on long-term patient-reported outcomes. We aimed to define multimorbidity patterns in a population of blast-injured military personnel, and to examine these patterns in relation to long-term quality of life (QOL).

**Methods:**

A total of 1972 US military personnel who sustained a blast-related injury during military operations in Iraq and Afghanistan were identified from clinical records. Electronic health databases were used to identify medical diagnoses within the first year postinjury, and QOL was measured with a web-based assessment. Hierarchical cluster analysis methods using Ward’s minimum variance were employed to identify clusters with related medical diagnosis categories. Duncan’s multiple range test was used to group clusters into domains by QOL.

**Results:**

Five distinct clusters were identified and grouped into three QOL domains. The lowest QOL domain contained one cluster with a clinical triad reflecting musculoskeletal pain, concussion, and mental health morbidity. The middle QOL domain had two clusters, one with concussion/anxiety predominating and the other with polytrauma. The highest QOL domain had two clusters with little multimorbidity aside from musculoskeletal pain.

**Conclusions:**

The present study described blast-related injury profiles with varying QOL levels that may indicate the need for integrated health services. Implications exist for current multidisciplinary care of wounded active duty and veteran service members, and future research should determine whether multimorbidity denotes distinct post-blast injury syndromes.

## Background

Following the terrorist attacks of September 11th, 2001, the US military initiated prolonged conflicts in Iraq and Afghanistan. The frontlines of battle were blurred, and enemy tactics focused on blast weaponry in the form of improvised explosive devices and other munitions [1]. A higher proportion of service members were injured by blasts during these conflicts than in any previous war [1–3]. Advances in combat casualty care, medical transport, and personal protective equipment led to a greater survivability from wounds, and the US Military Health System subsequently faced a growing number of service members and veterans with injury-related sequelae [3].

A robust body of literature now exists on blast injury, with most research conducted in the last 15 years. Blasts cause injury in a variety of ways, from the blast wave itself to fragmentation and blunt trauma [4]. The specific injuries resulting from blasts are numerous. In the Iraq and Afghanistan wars, concussions, serious extremity injuries, and amputations evidenced an unprecedented rise that paralleled the heightened use of blast weaponry [5–8]. Sensory injuries, such as hearing and vision deficits, have been linked to blasts as well [9–12]. Blasts can cause injuries to multiple systems, as one study among blast-injured military personnel found an average of nearly four injuries per patient [4]. The effects of blast move well beyond the physical, as the nature and circumstances of blast injury can often lead to psychological problems such as posttraumatic stress disorder (PTSD) and depression [13–15].

Multimorbidity describes a state where two or more medical conditions exist simultaneously in an individual, which can complicate treatment options and create the need for a diversity of health services [16]. Most of the research on multimorbidity in civilian populations has focused on older or chronically ill individuals [17–19]. This concept has also been explored in some recent military studies [20–23]. The polytrauma clinical triad (PCT) refers to three comorbid diagnoses of postconcussion syndrome, chronic pain, and PTSD [20]. A Department of Veterans Affairs (VA) study found PCT in 6% of veterans from the Iraq and Afghanistan conflicts [21]. Another study extended PCT to what they called “complex comorbidity clusters,” finding six unique clusters, including: PCT; PCT with chronic disease; mental health and substance abuse; sleep, amputation, and chronic disease; pain and moderate PTSD; and relatively healthy. Walker et al. described a similar idea, termed postdeployment multisymptom disorder (PMD), and suggested the need for an integrated treatment model in these cases [22]. It is important to note, however, that PCT describes a group of comorbid diagnoses postinjury, whereas PMD refers to symptomatology after deployment that may or may not include deployment-related injury.

The concept of a multisymptom disorder following deployment is not new; numerous studies have examined this phenomenon in veterans of the Gulf War, commonly referred to as Gulf War illness or chronic multisymptom illness (CMI). Further, the VA reports that CMI may also be common among veterans from the Iraq and Afghanistan conflicts [24]. Nevertheless, there is a paucity of literature on multimorbidity among service members with diagnosed blast-related injury, and the impact of these multiple disorders on postinjury patient-reported outcomes, such as quality of life (QOL). Patient-reported outcomes provide vital information about an individual’s subjective experience that cannot otherwise be captured via objective medical records. QOL in particular encompasses perceptions of one’s own mental, physical, and emotional status, as well as how well an individual is functioning in their everyday life [25]. Outcomes of blast-related injury can frequently result in activity limitation, participation restriction, and other reduced functioning, which may detrimentally affect QOL [26–28]. It is important to characterize multimorbidity resulting from blast-related injury, and to understand how it impacts patient-reported QOL in the military population. The purpose of this retrospective study was to assess whether unique clusters of multimorbidity exist following blast-related injury, and to describe these clusters by QOL.

## Methods

### Participants

This study included a subset of participants in the Wounded Warrior Recovery Project (WWRP). The WWRP longitudinally tracks patient-reported outcomes of active duty and veteran service members of the US military who were injured during a combat deployment and were provided care by military medical personnel [29]. Individuals were eligible for recruitment and enrollment in the WWRP through inclusion in the Expeditionary Medical Encounter Database (EMED), a US Navy-maintained deployment health database that contains service members’ clinical encounters while deployed [30]. Recruitment and data collection, which began in 2012, are ongoing and conducted on a rolling basis. To date, 37,455 individuals have been invited once (i.e., mailed letters) to participate in the WWRP and less than 1% of those invited have actively declined (*n* = 37). Participants are asked to complete a web-based baseline assessment battery and follow-ups every 6 months for 15 years. The IRB protocol allows for multiple recruitment attempts, thus, many of those invited who have not yet enrolled will be re-contacted prior to being considered “passively declined.” There are currently 6261 participants in the WWRP. Further details of the WWRP infrastructure and current methodology are described elsewhere [29].

The current study presents baseline patient-reported data for 1972 participants who were identified as having a blast-related injury between February 2003 and August 2014 after detailed review of their EMED clinical record, and had baseline WWRP assessments conducted between November 2012 and April 2017. The median time between injury and WWRP survey response was 6.4 years (interquartile range = 3.3–9.2). All WWRP procedures have received Naval Health Research Center Institutional Review Board approval.

### Measures

#### Demographic and service-related characteristics

Demographic and service-related variables at the time of injury were extracted from the Defense Manpower Data Center. Demographic variables included sex and age (18–24, 25–29, or 30+ years). Service-related variables included service branch (Army, Marine Corps, or Other) and rank/paygrade (enlisted [E1–E3, E4–E6, or E7–E9] or warrant officer/officer).

#### Injury-related characteristics

Information from the EMED was extracted to characterize the severity and date of injury. Injury severity was quantified using the Abbreviated Injury Scale (AIS) and Injury Severity Score (ISS). AIS classifies the severity of each injury by nine body regions from 1 (*minor*) to 6 (*currently untreatable*) [31]. ISS is derived from the AIS and assesses overall trauma severity with a focus on mortality risk, and is useful for quantifying the impact of multiple injuries [32]. The ISS is derived from the sum of squares of the highest AIS code in each of the three most severely injured body regions and ranges from 0 (*no injury*) to 75 (*fatal injury*). Injuries can be categorized based on ISS: 1–3 (*minor injury*), 4–8 (*moderate injury*), 9–15 (*serious injury*), 16–24 (*severe injury*), and 25 or greater (*critical injury*) [33, 34].

#### Physical and mental health diagnoses

Physical and mental health diagnoses from the first year postinjury (i.e., 1–365 days after injury) were extracted from medical records data in the Military Health System Data Repository. Outpatient *International Classification of Diseases*, 9th Revision, Clinical Modification (ICD-9-CM) codes were used [35], and grouped into Clinical Classifications Software (CCS) categories [36]. The CCS, developed by the Agency for Healthcare Research and Quality, is a tool for grouping patient diagnoses and procedures into a manageable number of clinically meaningful categories. There are a total of 261 separate CCS categories, which include the entire range of health conditions (e.g., mental health disorders, physical ailments, and chronic diseases). To facilitate the cluster analysis, participants had binary variables coded for each CCS category to indicate presence or absence. Because this study examined multimorbidity, participants required a minimum of two ICD-9-CM codes in at least two of these CCS categories to be included in the study.

#### QOL

QOL was assessed with the Quality of Well-Being Scale, Self-Administered (QWB-SA), a preference-weighted measure that assesses overall QOL based on patients’ self-reported ratings of recent problems related to mobility, physical functioning, social activity, and chronic and acute symptoms and conditions [37, 38]. From the QWB-SA, a total score can be calculated that quantifies well-being on a continuum of 0 (*death*) to 1 (*asymptomatic full function*). The QWB-SA has been used in civilian [39–42], veteran [43], and previous WWRP samples [44]. Specifically, a previous WWRP sample had an estimated QWB-SA of 0.513 [44], and other QWB-SA estimates include 0.643 for general outpatients aged 31–40 [45], 0.492 for migraine sufferers [42], 0.479 and 0.383 for depressed outpatients and inpatients, respectively [40], and 0.369 for older veterans with severe PTSD [43]. Clinically meaningful differences in QWB-SA scores can also be estimated [46].

### Statistical analysis

SAS version 9.4 (SAS Institute Inc., Cary, NC) was used for all analyses. Cluster analysis was performed as per previous research on multimorbidity [47]. Hierarchical clustering was used to identify clinically relevant groups of sample participants with similar CCS categories. With hierarchical clustering, each sample participant starts as their own cluster and is merged with the most similar participant. The process continues until there is only one cluster containing all observations. Multiple algorithms are available for use in hierarchical clustering, and we selected Ward’s minimum variance method. Ward’s considers every possible cluster combination at each step of hierarchical clustering and selects the combination resulting in the smallest addition to the error sum of squares. Ward’s method minimizes the variance within clusters and is known to produce clusters of similar sizes [48]. For cluster selection, the dendrogram and pseudo T were examined to identify the ideal number of clusters. An inclusion criterion of 0.5 was used to determine relevant CCS categories for each cluster. Duncan’s multiple range test was used to further group clusters into domains using QOL. Identified domains and clusters were described by the percent of study sample, QOL, and ISS.

## Results

Descriptive characteristics of the study sample are shown in Table 1. Most participants were male (95.2%), E4–E6 (60.5%), in the Army (76.0%), and under age 30 years at time of injury (70.6%). The mean ISS for the overall sample was 7.6 (SD = 7.9), and mean QOL 0.476 (SD = 0.143).

**Table 1:** Characteristics of the study sample (*n =* 1972)

Characteristic	*n*	%
Age, years		
18–24	857	43.5
25–29	536	27.2
30+	579	29.4
Service branch		
Army	1499	76.0
Marine Corps	396	20.1
Other	77	3.9
Sex		
Female	94	4.8
Male	1878	95.2
Rank/pay grade		
E1–E3	339	17.2
E4–E6	1194	60.5
E7–E9	234	11.9
Warrant officer/officer	205	10.4

Based on the dendrogram and pseudo T, five clusters were selected. A total of 11 CCS categories met the 0.5 inclusion criteria in at least one cluster, and Table 2 provides a breakdown of these categories, with the most frequent ICD-9-CM codes shown. Table 3 details the CCS categories for the five clusters. The clusters were defined as musculoskeletal, heterogeneous, concussion/anxiety, polytrauma, and clinical triad. The musculoskeletal cluster was predominated by other connective tissue disorder and other joint disorder, which mostly consisted of muscle weakness and pain (see Table 2). The heterogeneous cluster was diverse, without any CCS meeting inclusion criteria. Intracranial injury, headache/migraine, and anxiety disorder were key components of the concussion/anxiety cluster. The polytrauma cluster contains other connective tissue disorder, other nerve disorder, other joint disorder, other injury, open wound extremities, and open wound head, neck, and trunk. The most CCS categories that met inclusion criteria were in the clinical triad cluster (i.e., other nerve disorder, other connective tissue disorder, other joint disorder, intracranial injury, headache/migraine, back problem, anxiety disorder, and adjustment disorder). The term clinical triad refers to the co-occurrence of musculoskeletal pain, concussion, and mental health morbidity.

**Table 2:** Top 3 *International Classification of Diseases*, 9th Revision, Clinical Modification (ICD-9-CM) diagnoses for primary Clinical Classifications Software (CCS) categories identified in cluster analysis

CCS category	Top 3 ICD-9-CM diagnoses (%)		
Adjustment disorders	Unspecified adjustment reaction (25.7)	Adjustment disorder with mixed anxiety and depressed mood (24.4)	Adjustment disorder with anxiety (23.8)
Anxiety disorders	Posttraumatic stress disorder (62.5)	Anxiety state, unspecified (26.1)	Other acute reactions to stress (5.8)
Back problem	Lumbago (43.4)	Cervicalgia (18.1)	Pain in thoracic spine (13.0)
Headache/migraine	Headache (35.2)	Other headache syndromes (30.1)	Posttraumatic headache, unspecified (10.7)
Intracranial injury	Late effect of intracranial injury without mention of skull fracture (41.8)	Intracranial injury of other and unspecified nature (24.9)	Concussion with loss of consciousness of 30 min or less (6.5)
Open wound extremity	Traumatic amputation of leg, unilateral, below knee (10.1)	Traumatic amputation of leg, unilateral, at or above knee (9.0)	Traumatic amputation of leg, bilateral (7.2)
Open wound head, neck, trunk	Open wound, multiple sites (17.1)	Open wound of buttock (10.3)	Open wound of ear drum (7.5)
Other connective tissue	Muscle weakness (37.3)	Pain in limb (29.1)	Myalgia and myositis (4.7)
Other joint disorder	Difficulty in walking (17.3)	Pain in joint, lower leg (16.1)	Pain in joint, ankle and foot (13.0)
Other nerve disorder	Abnormality of gait (44.9)	Lesion of ulnar nerve (4.3)	Other chronic pain (4.0)
Other injury	Posttraumatic wound infection (14.2)	Other specified sites, including multiple injury (11.3)	Head injury, unspecified (9.0%)

**Table 3:** Percent distribution of Clinical Classifications Software (CCS) categories by cluster

CCS category	Cluster				
	Musculoskeletal	Heterogeneous	Concussion/ anxiety	Polytrauma	Clinical triad
Adjustment disorders	11.4	17.8	32.4	24.1	52.9^*a*^
Anxiety disorders	13.3	21.8	53.7^*a*^	30.3	76.0^*a*^
Back problem	3.9	27.3	45.3	23.4	54.1^*a*^
Headache/migraine	4.6	11.9	83.8^*a*^	17.5	59.5^*a*^
Intracranial injury	7.3	12.1	52.1^*a*^	49.6	63.2^*a*^
Open wound extremity	40.4	12.6	3.2	67.5^*a*^	28.1
Open wound head, neck, trunk	14.7	9.5	2.9	54.7^*a*^	19.0
Other connective tissue	56.2^*a*^	11.0	14.9	74.1^*a*^	69.4^*a*^
Other joint disorder	72.4^*a*^	24.5	21.7	55.5^*a*^	72.3^*a*^
Other nerve disorder	27.6	14.0	39.5	77.4^*a*^	70.7^*a*^
Other injury	21.2	11.5	4.5	72.6^*a*^	21.1

^a^Met inclusion criterion of 0.5

Table 4 details the results of Duncan’s multiple range test, grouping the five clusters into domains based on QOL. Clusters were further described by percentage of study sample and ISS. The largest group was the heterogeneous cluster, which comprised 39.1% of the study sample. The highest ISS was in the polytrauma cluster and the smallest was in the concussion/anxiety cluster (17.1 vs. 4.0, respectively). The lowest QOL was in the clinical triad cluster, and the highest was in the musculoskeletal and heterogeneous clusters. Both the polytrauma and concussion/anxiety clusters had similar QOL.

**Table 4:** Duncan’s multiple range test grouping of clusters by quality of life (QOL)

Duncan group	Cluster	*n*	%	QOL	ISS
A	Musculoskeletal	518	26.3%	0.501	7.3
	Heterogeneous	629	31.9%	0.496	5.3
B	Concussion/anxiety	309	15.7%	0.459	4.0
	Polytrauma	274	13.9%	0.452	17.1
C	Clinical triad	242	12.3%	0.416	8.3

## Discussion

The primary finding of the present study was the identification of patient profiles following blast-related injury based on multimorbidity and QOL. These distinct medical clusters provide further evidence that modern day combat produces injuries with multifaceted outcomes requiring integrated treatment strategies. To our knowledge, this study represents the first attempt to quantify QOL among US military personnel based on their multimorbidity status. The cluster with the lowest QOL contained diagnoses that suggested a clinical triad consisting of musculoskeletal pain, concussion, and mental health morbidity, similar to the PCT described previously in the literature [20]. This suggests that unique post-blast injury syndromes may exist, which would benefit from strategic allocation of health services and multidisciplinary refinements to clinical practice guidelines.

Implications of the present findings may include the creation of a risk dashboard to distinguish vulnerable populations. The five identified clusters were grouped into three QOL domains. Based on the minimal difference in QWB-SA identified by Le et al., the domains were clinically distinct in regard to QOL [46]. It is notable that the multimorbidity clusters consisted of medical diagnoses within the first year postinjury and still appeared to impact QOL an average of 6 years later. Although additional research is needed, this finding suggests that the physical and mental health problems an individual experiences within the first year postinjury could act as an early warning matrix (e.g., clinical decision tree) for future medical and psychological status. Without effective treatment, these individuals may continue to experience many of these problems for years. Thus, targeting key health services and implementing optimized intervention strategies during this first year following injury may result in improved outcomes for years to follow.

Musculoskeletal pain was ubiquitous in many of the identified clusters. This finding was not surprising considering that musculoskeletal injury is highly prevalent in the military and confers a substantial contribution to disability among military members [49, 50]. Interestingly, although both the musculoskeletal and clinical triad clusters had similar ISSs, the latter cluster had substantially lower QOL that exceeded minimally clinically important differences [46]. The clinical triad cluster, characterized by musculoskeletal pain, concussion, and mental health morbidity, had the lowest QOL and affected 12.3% of the study population. Central pain mechanisms may be influenced by psychological factors or a result of concussion [51, 52]. Although impossible to delineate in this study, it is plausible that the combination of comorbid concussion, psychological disorders, and musculoskeletal pain contributes to greater real or perceived functional limitations relative to the other symptom clusters. Alternatively, it may also reflect a higher level of psychological trauma experienced at the time of injury.

There were a multitude of adverse health conditions observed in the clinical triad cluster. Specific diagnoses included concussion with associated symptomology (e.g., headache), anxiety and adjustment disorders, and pain in the joints, back, and connective tissue. This resembles the PCT identified in previous literature [20, 21]. The PCT, however, assumes polytrauma, which is typically defined as an ISS ≥16 [53]. In our clinical triad cluster, the mean ISS was half that. Therefore, in the circumstances of blast-related injury, a term such as “post-blast clinical triad” may be more appropriate than PCT. This altered terminology will extend the definition to include individuals with mild injuries who also experience comorbid musculoskeletal pain, concussion, and mental health issues. Further research is needed to determine whether this triad exists as a distinct entity (or syndrome), rather than the sum of individual conditions.

The results posit the question of whether unique post-blast injury syndromes may exist that can be defined by multimorbidity. As previously mentioned, there is a body of evidence supporting multisymptom disorders associated with wartime deployment. The most notable in recent history were Gulf War illness or CMI, terms used to describe a variety of health-related complaints of unknown etiology among Gulf War veterans [54, 55]. Previous military conflicts have described combat stress disorders that were frequently associated with both psychological and physical issues [56–58]. Concussion and PTSD have been linked to multiple comorbidities, including chronic pain, sleep problems, and other mental health disorders [59–61]. Because blasts can affect multiple systems simultaneously, the concept of unique post-blast injury syndromes is plausible [4].

Another finding of this study was that ISS did not appear to have a direct relationship with QOL. This is consistent with civilian literature, which has found that subjective, rather than objective, injury severity tends to be a better predictor of psychological morbidity [62, 63]. In fact, ISS was created as an indicator of mortality risk from the injury itself, not QOL [33]. It was also interesting that, despite vastly divergent ISS values, the concussion/anxiety and polytrauma clusters yielded similar QOL scores. This finding provides further support for the importance of what has been termed the “invisible wounds” of war such as concussion and PTSD, which can adversely affect health without physical signs [64]. Lastly, the heterogeneous cluster showed that 39.1% of the study sample did not have a clearly defined pattern of multimorbidity. This may speak to the overall burden of multimorbidity following blast-related injury, and suggests that a prevalence estimate for multimorbidity could be calculated based on future studies and agreed upon clinical criteria. Additional research is needed to determine whether this pattern of multimorbidity is consistent across other populations with blast-related injury or unique only to military personnel.

These findings underline the importance of multidisciplinary, integrated care across a variety of settings that treat active duty and veteran service members. While the military has progressively moved to a patient-centered medical home model, which employs a team-based approach for in-garrison managed care [65], additional measures to improve care for service members with multimorbidity may be warranted. Namely, inclusion of interdisciplinary rehabilitation, sports medicine, orthopaedic, and mental health specialists in deployed units may help with the prompt assessment, treatment, and disposition of patients with musculoskeletal pain, concussion, and mental health conditions [66, 67]. Collocation of interdisciplinary specialists with operational units help to mitigate perceived or actual barriers to care-seeking, factors that may contribute to long-term morbidity [68]. This recommendation, while salient for past conflicts, will be increasingly important in future conflicts where long latencies before a casualty is transported are anticipated [69].

Consistent with patient-centered care, routine assessment of patient-reported outcomes, including QOL, should be utilized when caring for military members following injury. Quantifying QOL throughout the healing process and during rehabilitation may provide critical information regarding outcomes of importance to the patient. Clinicians, regardless of specialty, should consider the whole patient for physical, psychological, and cognitive impairment when caring for this population. In addition to standard assessments that may be identified in a healthcare system, the Patient-Reported Outcomes Measurement Information System may also be a useful resource for identifying assessments for a variety and range of problems (e.g., depressed mood, sleep, and pain), and assessing health-related QOL based on changes in physical and mental health [70].

There are limitations to this analysis. The clustering method used is exploratory in nature and, thus, no causality can be inferred. Results should be replicated using a longitudinal and multivariate approach. Outpatient ICD-9-CM codes were only available for those who presented for care at a military treatment facility. As such, individuals who were self-treating or who received care outside of the Department of Defense may have been missed in the analysis. In addition, the QOL analysis was univariate only, and future studies should examine possible confounders in multivariable analysis. Further, there is potential for selection bias in this study in that participants may not be representative of the larger blast-injured population. Nevertheless, WWRP non-responders have been shown to be similar to responders across most variables with the exception of age (i.e., responders tend to be older) [44]. Lastly, the study period was long, and there may be relevant variables unaccounted for during the period between injury event and QOL measurement (e.g., changes in financial status, social support).

This study also had strengths that warrant mention. The use of the EMED allowed for the selection of a large sample of individuals with blast-related injuries and ISS from the point of injury. Although outpatient ICD-9-CM codes in this study have their inherent limitations (e.g., dependence on patient presenting for health services), their use in predicting long-term patient-reported outcomes such as QOL may prove useful for targeting clinical interventions. Categorizing ICD-9-CM codes in CCS made use of a validated method for analyzing large data sets. Using QOL as the outcome measure was a comprehensive way to assess long-term health status, and its multifaceted nature aligned appropriately with the focus on multimorbidity.

## Conclusion

Blast injury is an emerging public health problem of modern warfare, often associated with multiple adverse outcomes that can overlap. The concept of multimorbidity shifts focus from primary to co-occurring medical conditions, and may be useful in determining treatment strategies and allocation of health services. The present study identified multiple, distinct clusters of medical diagnoses within 1 year following blast injury, which were associated with differing levels of QOL. A clinical triad consisting of musculoskeletal pain, concussion, and mental health morbidity was present in a subset of patients and corresponded to a low QOL. More research is needed to highlight other acute and chronic effects of multimorbidity and refine clinical practice guidelines for blast-injured active duty and veteran service members.

## Data Availability

The datasets generated and/or analyzed during the current study are not publicly available due to personally identifiable information regulations, but may be made available by the corresponding author on reasonable request and approval by the Naval Health Research Center Institutional Review Board.

## Abbreviations

AIS: Abbreviated Injury Scale
CCS: Clinical Classifications Software
CMI: Chronic multisymptom illness
EMED: Expeditionary Medical Encounter Database
ICD-9-CM: *International Classification of Diseases*, 9th Revision, Clinical Modification
ISS: Injury Severity Score
PCT: Polytrauma clinical triad
PMD: Postdeployment multisymptom disorder
PTSD: Posttraumatic stress disorder
QOL: Quality of life
QWB-SA: Quality of Well-Being Scale, Self-Administered
WWRP: Wounded Warrior Recovery Project
US: United States
